# Rasch Analysis of the QuickDASH in Patients with Neck Pain

**DOI:** 10.3390/jcm14061870

**Published:** 2025-03-11

**Authors:** Yen-Mou Lu, Yuh-Yih Wu, Yi-Jing Lue

**Affiliations:** 1 Department of Orthopaedics, School of Medicine, College of Medicine, Kaohsiung Medical University, Kaohsiung 807, Taiwan; yemolu@kmu.edu.tw; 2 Division of Pediatric and Spinal Orthopedics, Department of Orthopedics, Kaohsiung Medical University Hospital, Kaohsiung 807, Taiwan; 3 Department of Special Education, National Kaohsiung Normal University, Kaohsiung 802, Taiwan; t1850@nknucc.nknu.edu.tw; 4 Department of Physical Therapy, College of Health Sciences, Kaohsiung Medical University, Kaohsiung 807, Taiwan; 5 Department of Medical Research, Kaohsiung Medical University Hospital, Kaohsiung 807, Taiwan

**Keywords:** neck pain, Rasch analysis, QuickDASH, function, symptom

## Abstract

**Background/Objectives**: Many patients with neck pain have arm problems. The purpose of this study was to examine the psychometric properties of the QuickDASH in patients reporting neck pain by Rasch analysis. **Methods**: The study was a cross-sectional study. Rasch analysis was used to examine the QuickDASH for unidimensionality, category function, item difficulty and targeting, and reliability in patients with neck pain. The two-factor model, comprising a function factor (items 1–8) and symptom factor (items 9–11), were separately assessed by Rasch analysis. **Results**: The mean age of the 302 participants was 57.9 ± 10.4 years old. The mean QuickDASH score was 24.8 ± 23.3 (95% CI: 22.2–27.5). For the function factor, the InfitMNSQ/OutfitMNSQ ranges were 0.700–1.124/0.661–1.121, indicating that all items fitted the model’s expectation. Only two items (items 4 and 6) exhibited category response disorder. The map for the person–item response thresholds covered the patient distribution well. The reliability was good, with a person separation index of 0.85. For the symptom factor, the InfitMNSQ/OutfitMNSQ ranges were 0.522–0.863/0.517–0.885, indicating that all items fitted the model’s expectation. No items with category response disorder were found for the symptom factor, and the reliability was good, with a person separation index of 0.82. **Conclusions**: The items fit the Rasch model well, and the wide range of item response thresholds covered the ranges of the patients’ disability and symptoms well. The QuickDASH has a two-factor structure, and is an excellent measure of upper limb function and symptoms in patients reporting neck pain.

## 1. Introduction

Neck pain is a common musculoskeletal problem. In about 80% of patients with neck pain, upper limb activities aggravate the pain, and higher neck pain severity is associated with greater upper limb functional restriction [1]. In such patients, the symptoms and disability of the upper limb need to be assessed; however, no suitable neck instruments assess upper limb problems. The Quick Disability of the Arm, Shoulder, and Hand (QuickDASH) is an abbreviated measure of physical function and symptoms in patients with upper limb disorders, developed from the DASH by Beaton et al. in 2005 [2]. It has good content and psychometric properties, and is commonly used for assessing patients with arm–shoulder–hand disorders [3]. However, few studies have examined the psychometric properties of the QuickDASH in a group of patients reporting neck pain.

The psychometric properties of an instrument should be validated by both classical test theory (CTT) and modern test theory (MTT). The former has disadvantages that can be overcome by MTT. For example, in CTT, a measure with more items is more reliable than a measure with fewer items to test a latent trait, and the score of each item is ranked the same, without considering item difficulty [4]. In MTT, Rasch modeling can test whether the items in an instrument form a single construct (unidimensionality) [5]. If not, some items measure different traits, and possibly other constructs [6]. Rasch modeling can also transform ordinal scores into interval scores on a logit scale [7], overcoming the scoring problems of CTT. Rasch modeling also provides the order of item difficulty, the precision of the disability estimates, and the appropriacy of the response categories for each item.

A few studies have used Rasch modeling to examine the psychometric properties of the QuickDASH, with inconsistent results [8,9,10]. Rasch analysis has identified problems that cannot be easily detected using traditional analyses in patients with shoulder pain [9]. Franchignoni et al. examined 283 patients with upper limb disorder and found that item 10, “tingling”, did not fit the Rasch model [8]. A two-factor model (task and symptom) of the QuickDASH provided a high-quality, continuous, and condition-specific scale for patients with carpal tunnel syndrome after surgery [10]. To date, no studies have assessed the use of the QuickDASH in patients with neck pain with Rasch modeling. Therefore, the purpose of this study was to explore the structural validity of the QuickDASH by MTT Rasch analysis in patients with neck pain to test unidimensionality, explore the order of item difficulty on an interval scale using logits, evaluate the category responses, and assess reliability. The hypothesis of this study was that the QuickDASH would fit the Rasch model well in patients with neck pain, with a two-factor model having ordered response categories and good reliability.

## 2. Materials and Methods

### 2.1. Participants

**Figure 1:**
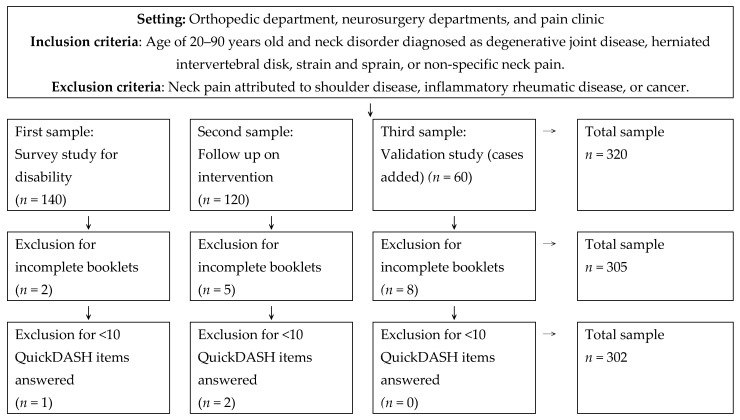
Consecutive samples diagram.

The study was a cross-sectional study. This retrospective study used data from two prospectively collected databases. The study included participants aged 20 to 90 years. Patients with neck pain, with or without arm pain, were recruited from outpatients and inpatients of the orthopedics department, the neurosurgery department, and the pain clinic of Kaohsiung Medical University Hospital in Kaohsiung, Taiwan, from April 2011 to May 2024. Patients identified according to symptoms, physical signs, and imaging study results were recruited by physicians. The causes of the neck pain were variously diagnosed as degenerative joint disease, herniated intervertebral disk, strain/sprain, and non-specific etiology. Participants with neck pain caused by shoulder disease, inflammatory rheumatic disease, or cancer were excluded. The consecutive samples diagram is shown in Figure 1. We excluded data from patients who answered fewer than 10 items on the QuickDASH, as its instructions require a minimum of 10 out of 11 items to be completed. As a result, three participants were removed. If a participant completed only 10 items, we did not impute the missing data using statistical methods. Ultimately, 302 patients with neck pain, with or without arm pain, were included in the Rasch analysis. The study received approval from the hospital’s Institutional Review Board (KMUH-IRB-980589 and KMUH-IRB-E(I)-20210363), and all participants gave written informed consent. The QuickDASH was used to assess the upper limb problems in all participants.

### 2.2. Instrument

The QuickDASH contains 11 items, as follows: opening a jar (item 1); performing housework (item 2); holding a shopping bag (item 3); reaching behind oneself to wash one’s back (item 4); manipulating a knife (item 5); recreational activities with force or impacts (item 6); interference with social activities (item 7); limitations in work or daily activities (item 8); shoulder, arm, or hand pain (item 9); tingling (item 10); and difficulty sleeping (item 11). The two optional modules (work and sports/performing arts) were not used in this study. Each item is scored on a 5-point scale, with 1 representing no limitation and 5 representing maximal limitation. For valid score calculation, a minimum of 10 of the 11 items must be completed.

### 2.3. Rasch Analysis

In Rasch measurement, the rating scale model assumes that all items share the same rating scale structure, while the partial credit model allows each item to have its own rating scale structure. Since the items of the QuickDASH do not share a uniform rating scale structure, the partial credit model for polytomous items was selected for Rasch analysis [8,9]. The two-factor model of the QuickDASH for patients with neck pain has been supported by exploratory factor analysis (EFA) and confirmatory factor analysis (CFA) [11]. The two factors are the function factor (items 1–8) and the symptom factor (items 9–11). Therefore, separate Rasch analyses were performed for each factor. The analyses were conducted in IBM’s SPSS software version 27, Extend Rasch (R version 3.6), as follows: (1) The responses of the items were coded as 01234 scores according to the format of extend Rasch; (2) the unidimensionality and category function were examined; (3) if an item was misfit, the item was deleted, and if category response disorder was detected, the responses were recoded; and (4) the Rasch analysis was repeated.

#### 2.3.1. Unidimensionality

Whether the data fit the model’s expectations was inspected with infit and outfit statistics. The former are sensitive to unexpected responses on items close to patient’s measured level, and the latter are sensitive to unexpected responses far from the person’s measured level [12]. The weighted mean square of the infit mean square (InfitMNSQ) and unweighted mean square of the outfit mean square (OutfitMNSQ) were employed to indicate the fit of the model. The acceptable ranges of InfitMNSQ and OutfitMNSQ are between 0.5 and 1.5 [13].

#### 2.3.2. Category Function

For the determination of the appropriacy of the response categories, the probability category curves for each item were plotted to examine the category function. Each curve indicated the difficulty (x-axis) and the probability (y-axis) of a single response category. The intersections of successive category probability curves indicated the step difficulty (threshold). A response category design that is satisfactory has a monotonic progression of step difficulties [14,15]. A step difficulty was considered to be disordered if the difficulty of a step with a higher number was lower than its preceding step with a lower number. Disordering of the step difficulties necessitates adjustment of the response categories.

#### 2.3.3. Item Difficulty and Targeting

To identify how challenging the activities were for a patient to perform, the item difficulties were calculated and expressed in logits. How consistently the distribution of QuickDASH items aligned with the disability with the functional limitations of the patients was examined with targeting. Person–item response thresholds were plotted for the assessment of targeting, and any notable gaps in the item response thresholds were noted.

#### 2.3.4. Reliability

The test information function and person separation index (PSI) were used for reliability. The PSI classifies individual abilities by the items, with PSI values over 0.75 indicating good reliability [13]. To identify the information provided by the test, the test information curve was plotted. The inverse square root of the test information is the standard error (SE) of the Rasch person measure. Each individual item of information is summed to produce the test information. The test information curve provides a visual representation of test information based on each person’s ability. The quantity of information provided by a test is often referred to as the estimated precision [16]. The definition of precision in the present study was an SE smaller than 0.5, the corresponding value of information was >4, and reliability was indicated by values > 0.75, respectively [16]. Sensitivity analysis was performed by excluding missing data. First, participants with missing data were omitted. Then, the Rasch analysis was rerun without missing data, and the PSI results were compared.

## 3. Results

### 3.1. Demographic Data

After exclusion of the participants with incomplete booklets (*n* = 15) and those answering fewer than 10 of the QuickDASH items (*n* = 3), a total of 302 patients were included in the study (Figure 1). The proportion of missing data was 5.6%. The demographic data of the participants are listed in Table 1. Females (*n* = 174) outnumbered males (*n* = 128). About 25% were elderly (age ≥ 65), and over half were aged 50 to 64 years (56%). Most (80.2%) had chronic pain with disease durations exceeding 3 months. Neck pain severity varied from mild to severe, with a mean VAS value of 3.9 ± 2.9. Arm pain was scored slightly lower than neck pain, with a mean VAS value 3.6 ± 3.0.

### 3.2. Rasch Analysis for the Function Factor

**Figure 2:**
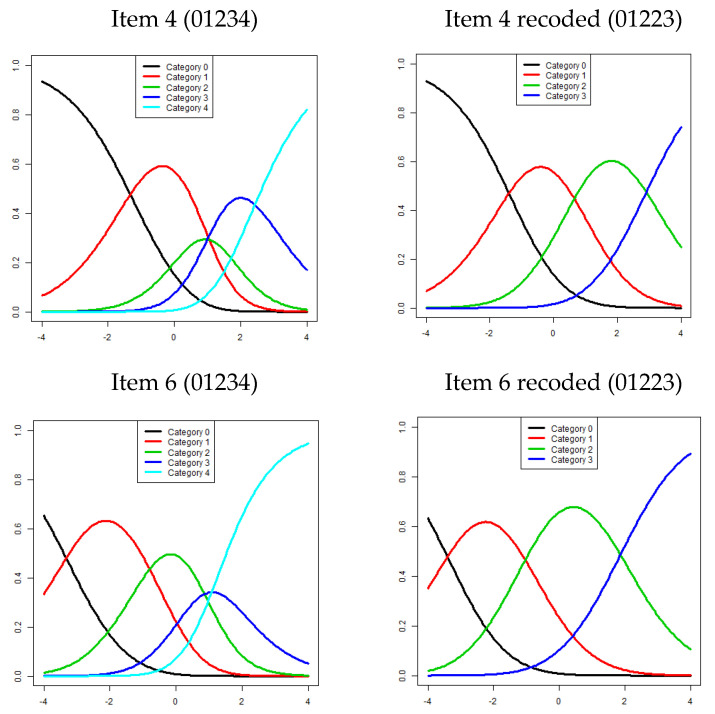
The response category probability curves for items 4 and 6. Left upper panel shows item 4 without recoding (01234) and right upper panel shows item 4 with recoding (01223); the left lower panel shows item 6 without recoding (01234) and the right lower panel shows item 6 with recoding (01223). After recoding, items 4 and 6 show well-functioning category responses. X-axis: item difficulty, Y-axis: category probability.

The InfitMNSQ of eight items ranged from 0.700 to 1.124, and the OutfitMNSQ ranged from 0.661 to 1.121, both falling within the acceptable range of 0.5–1.5. Those values showed that eight items (items 1–8) fitted the model’s expectations, indicating a unidimensional scale. Two items exhibited disordering in step difficulty (Figure 2). The numbers of response categories with disordered items were checked, and the response categories of two adjacent items were combined. The original coding of 01234 was recoded to 01223 (items 4 and 6). After further Rasch analysis, the step difficulties for each item indicated monotonic increases in the thresholds. The category functions of the other items (items 1, 2, 3, 5, 7, and 8) had well-functioning responses. Table 2 presents the InfitMNSQ/OutfitMNSQ and the step difficulties of each item after the final Rasch analysis.

Table 2 also lists the item difficulty of each item. The item with the highest difficulty (1.845 logits) was item 5, which measured disability using a knife. The one with the lowest item difficulty (−0.858 logits) was item 6, measuring disability in recreational activities with force or impacts.

**Figure 3:**
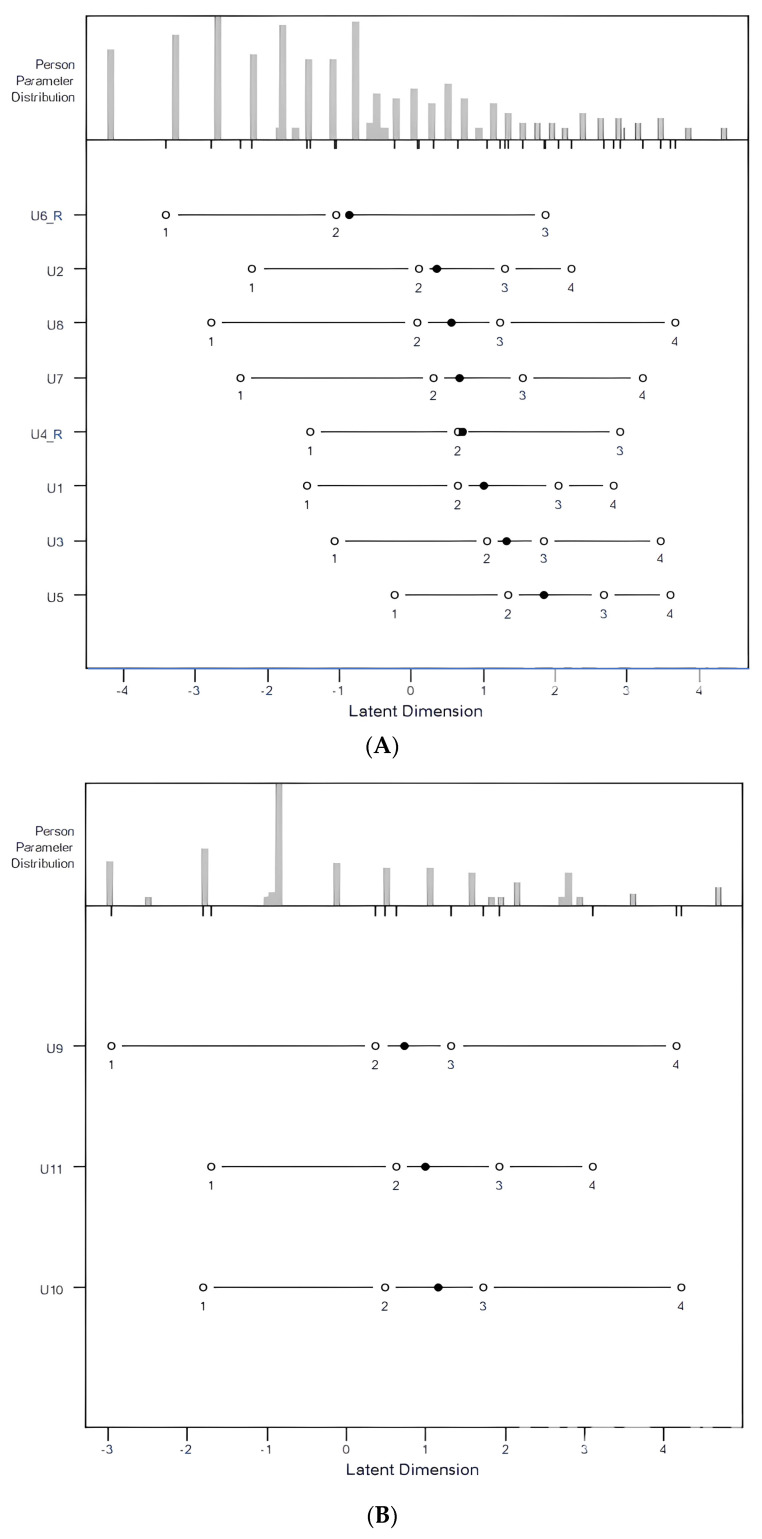
Person–item threshold map of the QuickDASH. (**A**) Items 1 to 8. (**B**) Items 9 to 11. The upper panel shows the person parameter distribution, and the lower panel shows the item difficulty. Items are sorted by item difficulty. X-axis: logits.

Person–item response thresholds are shown in Figure 3. The upper part is the person parameter distribution (in logit units), representing the frequency distribution of patients with different disabilities; the lower part shows the item difficulty distribution, and the thresholds of item categories are plotted, respectively. The range of the item response thresholds was 7.086 logits (−3.404 to 3.682), which covered the patient distribution evenly; no gaps were identified.

**Figure 4:**
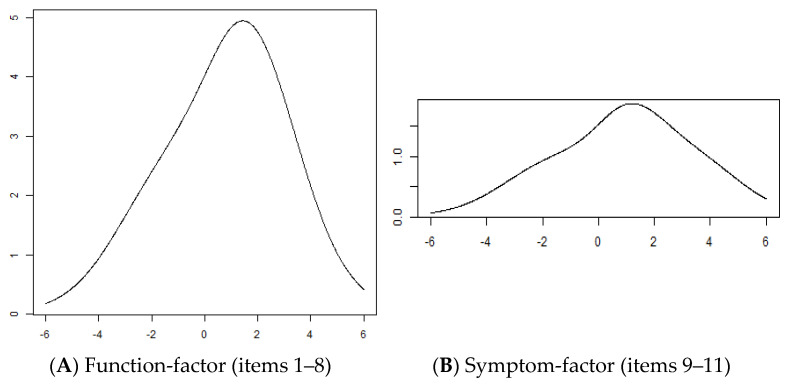
Test information curves of the QuickDASH. (**A**) For items 1 to 8. (**B**) For items 9 to 11. X axis: person logits.

The test information curve is presented in Figure 4A. The test information curve was in the form of a bell, with the maximum located at the middle of the person measure scale. The results showed that the value of the maximum test information was 4.94. The reliability was good, with a person separation index of 0.85 (a value above 0.75 indicates good reliability). The results of the sensitivity analysis were consistent with the findings (without excluding the missing data).

### 3.3. Rasch Analysis for the Symptom Factor

Table 3 lists the InfitMNSQ/OutfitMNSQ and the step difficulties of each item. The InfitMNSQ of the items were 0.522–0.863, and the OutfitMNSQ of the items were 0.517–0.885, respectively. Both InfitMNSQ and OutfitMNSQ fell within the acceptable range of 0.5–1.5. Those values showed that items 9–11 fitted the model’s expectations, indicating a unidimensional scale. The category function showed that all three items had well-functioning responses. The item difficulties of items 9–11 were 0.721, 1.154, and 0.990, respectively. Person–item response thresholds are shown in Figure 3B. The range of the item response thresholds was 7.179 logits (−2.966 to 4.213). The test information curve is shown in Figure 4B. Based on only three items, the value of the test information was low (2.22). The reliability was good, with a person separation index of 0.82 (a value above 0.75 indicates good reliability).

## 4. Discussion

The QuickDASH has two factors for patients with neck pain, namely, the function factor (items 1–8) and the symptom factor (items 9–11). No misfit items were found, and the unidimensionality of each was confirmed by Rasch analysis. Only two items (items 4 and 6) had category response disorder, which was solved by recoding. The range of the item response thresholds was wide, matching the person abilities for both the function factor and the symptom factor.

Our previous study used EFA to explore the factor structure and identify the two-factor model; after the EFA, CFA confirmed the two-factor structure [11]. In this study, Rasch analysis was applied separately for the function and symptom factors, and both fitted the Rasch model. In a study of the QuickDASH applied in patients with various upper limb disorders, the 11 items were not unidimensional [8]. The QuickDASH has also exhibited multidimensionality and significant misfit in patients with shoulder pain [9]. However, the two-factor model of the QuickDASH (task items 1–6 and symptom items 9–11) was examined in patients with carpal tunnel syndrome, and each factor fitted the Rasch model [10]. Therefore, in patients with neck pain and/or in patients with carpal tunnel syndrome, the QuickDASH may have a two-factor model.

Category response disorder is a common problem in instruments with multiple category responses. For example, in the Oswestry Disability Index, with six responses for each item, many items exhibited category response disorder [17]. Category response disorder in the QuickDASH was also found in patients with various upper limb problems. All 11 items were disordered in patients with upper limb dysfunction [8], and all 6 task items were closed or disordered in patients with Dupuytren disease [10]. Only two items (item 4: washing one’s back; item 6: recreational activities with force or impacts) with mild category response disorder were found in our study. Therefore, the category response disorder of the QuickDASH appears to be better in patients with neck pain than in patients with various upper limb problems.

The wide range of the item response thresholds covered the range of disability in patients with neck pain well. The ranges of the item response thresholds were 7.086 logits (−3.404 to 3.682) and 7.179 logits (−2.966 to 4.213) for the function and symptom factors, respectively (Figure 3). Jerosch-Herold et al. reported that the person–item threshold map of the QuickDASH had a high degree of overlap between person ability and item difficulty, with a difficulty spread within −4 to +3 logits in patients with shoulder pain [9]; however, a gap existed on the easier item end. Our study also found good overlap between person ability and item difficulty. Although the items for the symptom factor also fitted well, a minimum of three items is the lowest requirement for an instrument; therefore, the test information provided by individual items was low. We suggest that more items for the symptom factor be developed in the future, such as for patients with radiculopathy or myelopathy, as they may present with loss of sensation, muscle weakness, and other symptoms [18].

No items of the QuickDASH need to be deleted for application in patients with neck pain. Thus, the items of the QuickDASH might be more suitable for patients with neck pain than for patients with certain upper limb problems. For example, past studies have suggested deleting item 10 (tingling in arm, shoulder, or hand) due to only about 10% of patients reporting paresthesia [9] or due to underfitting (InfitMNSQ = 1.62; OutfitMNSQ = 2.01) [8]. However, item 10 fitted well in the symptom factor in patients with neck pain. Since tingling and radiculopathy are common in such patients, this item should not be deleted for application in patients with neck pain.

The clinical implication of the present study is that the QuickDASH provides excellent items for assessing arm function/symptoms in patients with neck pain. The items of the QuickDASH cover the person ability well, and the results support a previous validity study of the measure [11]. In this study, the QuickDASH function factor provided high test information, with the results supporting the excellent reliability of the measure in patients with neck pain [11]. Larger logits for item difficulty indicate greater disability/severity in patients with neck pain. For example, the largest item difficulty of the eight functional items was item 5 (difficulty using a knife), with 1.845 logits. A response indicating that a patient is unable to use a knife to cut food implies significant disability of the arm. Similarly, for the symptom items, the highest item difficulty was item 10 (tingling severity), with 1.154 logits. A response indicating extreme tingling (pins and needles) in the arm indicates high severity of the symptom and implies possible radiculopathy or myelopathy.

A study on the effects of a therapeutic exercise protocol for patients with chronic non-specific back pain reported that 34.9% of participants had both neck and back pain [19]. The study examined pain levels (mild, moderate, and severe) and disability levels (none, mild, moderate, severe, and complete). Pain and disability assessments are essential for clinical practice, and it is also important to evaluate neck pain in patients with low back pain. In this study, the QuickDASH was assessed for 302 patients with neck pain. The mean neck pain level was not severe (VAS = 3.9). According to a study on VAS cut-off points, pain levels are classified as mild (<3.5), moderate (3.5–7.4), and severe (>7.5) [20]. We examined QuickDASH values for patients with different neck pain severity levels. The QuickDASH score was lower in those with mild neck pain (14.9 ± 14.9) compared to those with moderate and severe neck pain (35.6 ± 25.8) (*p* < 0.001). In clinical practice, patients with moderate to severe neck pain may often experience persistent upper limb pain and disability, while those with mild neck pain may still have some degree of upper limb pain or disability.

Some limitations of our study should be noted. First, the sample size of the study was not large (*n* = 302). However, the Rasch model is a one-parameter logistic model, and a sample size of over 100 respondents is recommended [21]. According to the COSMIN study design suggestions, a sample size ≥ 200 is very good for a Rasch model [22]. The inclusion of even more cases could be useful in recognizing specific upper limb problem patterns associated with different diagnoses of neck problems. Second, the sample selection criteria may have incurred potential sampling bias. The participants with neck pain we included in the study were limited only to those with degenerative joint disease, herniated intervertebral disk, strain and sprain, and non-specific neck pain. Thus, the sample did not include patients with other causes of neck pain. It follows that the findings may not be generalizable to all cases of patients with various etiologies. Third, the different statistics software programs provide different types of reports, which may increase the difficulty of study comparisons. Finally, no subgroup analyses, such as analysis of patients with different diagnoses or differences in pain severity, were conducted in the study. Rasch analysis with a small sample entails a larger standard error, which leads to less precise estimates. Subgroup analysis was omitted due to an insufficient number of cases; however, such analysis in a larger sample could provide more information for clinical application.

## 5. Conclusions

The QuickDASH is an excellent instrument for assessing upper limb function and symptoms in patients with neck pain. It has a two-factor structure, with items 1–8 belonging to the function factor and fitting well, and items 9–11 belonging to the symptom factor and fitting well without category response disorder. In the Rasch analysis, the item difficulty and person ability were transformed into continuous scales by logit mathematics methods. The wide range of the item response thresholds well covered the ranges of the patients’ disability and symptoms.

## Figures and Tables

**Table 1:** Demographic characteristics of the participants.

Characteristic	Mean ± SD (Range) Percentage
Age (years)	57.9 ± 10.4 (21–84)
21–49	18.9%
50–64	56.0%
65–84	25.1%
Gender	
Male	42.4%
Female	57.6%
Disease duration (month)	54.2 ± 79.8 (0.3–600)
Acute (<6 wk)	7.8%
Subacute (6 wk–3 mo)	12.0%
Chronic (>3 mo)	80.2%
Education	
Elementary school	23.4%
High school	52.2%
University	24.4%
Marital status	
Single/widowed	16.1%
Married	83.9%
Occupation	
Office worker	23.3%
Laborer	22.4%
Homemaker	22.4%
Unemployed	5.7%
Retired	26.2%
Diagnosis	
Degenerative joint disease	37.9%
Herniated intervertebral disk	29.0%
Strain/sprain	10.6%
Non-specific neck pain	22.5%
VAS score	
Neck	3.9 ± 2.9 (0–10)
Arm	3.6 ± 3.0 (0–10)

**Table 2:** Item difficulty, fit statistics, and category function of item 1 to item 8 after recoding of items 4 and 6.

Item	Difficulty Logit	InfitMNSQ	OutfitMNSQ	Threshold 1	Threshold 2	Threshold 3	Threshold 4
1: Difficulty opening a jar	1.016	1.124	1.121	−1.447	0.653	2.038	2.818
2: Difficulty performing household chores	0.359	0.747	0.757	−2.206	0.108	1.297	2.237
3: Difficulty carrying a shopping bag	1.321	0.774	0.821	−1.061	1.049	1.836	3.461
4: Difficulty washing your back	0.721	1.017	1.081	−1.401	0.652	2.911	NA
5: Difficulty using a knife	1.845	0.700	0.661	−0.237	1.348	2.668	3.601
6: Difficulty in recreational activities	−0.858	0.955	0.934	−3.404	−1.035	1.866	NA
7: Interference with social activities	0.671	0.938	0.893	−2.378	0.307	1.544	3.211
8: Limitation in work/daily activities	0.551	0.857	0.839	−2.787	0.074	1.235	3.682

InfitMNSQ: infit weighted mean square; OutfitMNSQ: outfit mean square; Threshold: the measure’s ability in the equal probability of two successive responses. Threshold 1 (categories between 1 and 2), threshold 2 (categories between 2 and 3), threshold 3 (categories between 3 and 4), and threshold 4 (categories between 4 and 5). After recoding of item 4 and item 6, all items showed a monotonic increase in the thresholds.

**Table 3:** Item difficulty, fit statistics, and category function of items 9 to 11.

Item	Difficulty Logit	Infit MNSQ	Outfit MNSQ	Threshold 1	Threshold 2	Threshold 3	Threshold 4
1: Pain severity	0.721	0.522	0.517	−2.966	0.372	1.327	4.153
2: Tingling severity	1.154	0.602	0.603	−1.802	0.478	1.727	4.213
3: Difficulty sleeping	0.990	0.863	0.885	−1.700	0.630	1.926	3.106

InfitMNSQ: infit-weighted mean square; OutfitMNSQ: outfit mean square; Threshold: the measure’s ability in the equal probability of two successive responses. Threshold 1 (categories between 1 and 2), threshold 2 (categories between 2 and 3), threshold 3 (categories between 3 and 4), and threshold 4 (categories between 4 and 5). All items showed a monotonic increase in the thresholds.

## Data Availability

The datasets generated and analyzed during the current study are available from the corresponding author upon reasonable request.

## Abbreviations

The following abbreviations are used in this manuscript:

QuickDASH	Quick Disability of the Arm, Shoulder, and Hand
InfitMNSQ	Weighted mean square of the infit mean square
OutfitMNSQ	Unweighted mean square of the outfit mean square
